# PAF-Wnt signaling-induced cell plasticity is required for maintenance of breast cancer cell stemness

**DOI:** 10.1038/ncomms10633

**Published:** 2016-02-04

**Authors:** Xin Wang, Youn-Sang Jung, Sohee Jun, Sunhye Lee, Wenqi Wang, Andrea Schneider, Young Sun Oh, Steven H. Lin, Bum-Joon Park, Junjie Chen, Khandan Keyomarsi, Jae-Il Park

**Affiliations:** 1 Department of Experimental Radiation Oncology, University of Texas MD Anderson Cancer Center, 6565 MD Anderson Boulevard, Z6.6034, Unit 1052, Houston, Texas 77030, USA; 2 Department of Molecular Biology, Pusan National University, Busan 609-735, Korea; 3 Program in Genes and Development, University of Texas MD Anderson Cancer Center, Houston, Texas 77030, USA; 4 Graduate School of Biomedical Sciences, University of Texas Health Science Center and MD Anderson Cancer Center, Houston, Texas 77030, USA

## Abstract

Cancer stem cells (CSCs) contribute to tumour heterogeneity, therapy resistance and metastasis. However, the regulatory mechanisms of cancer cell stemness remain elusive. Here we identify PCNA-associated factor (PAF) as a key molecule that controls cancer cell stemness. PAF is highly expressed in breast cancer cells but not in mammary epithelial cells (MECs). In MECs, ectopic expression of PAF induces anchorage-independent cell growth and breast CSC marker expression. In mouse models, conditional *PAF* expression induces mammary ductal hyperplasia. Moreover, PAF expression endows MECs with a self-renewing capacity and cell heterogeneity generation via Wnt signalling. Conversely, ablation of endogenous PAF induces the loss of breast cancer cell stemness. Further cancer drug repurposing approaches reveal that NVP-AUY922 downregulates PAF and decreases breast cancer cell stemness. Our results unveil an unsuspected role of the PAF-Wnt signalling axis in modulating cell plasticity, which is required for the maintenance of breast cancer cell stemness.

## Abstract (web summary)

Stem cells are found in many tumour types and are thought to be partially responsible for cell survival following therapy. Here, the authors show that PCNA-associated factor, PAF, contributes to stemness in breast cancer cells and pharmacological targeting of PAF reduces mammosphere formation.

## Graphical abstract

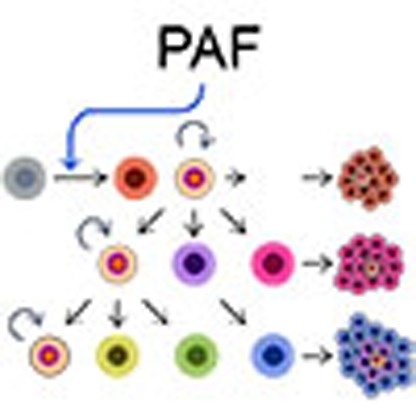

Stem cells (SCs) are characterized by their abilities to self-renew and to constitutively or conditionally differentiate into committed cells^1^. Cellular heterogeneity driven by SCs is tightly controlled by developmental signalling pathways during development and regeneration. In recent times, an emerging concept, ‘cell plasticity', has challenged the paradigm that SCs are the source of cell heterogeneity. In the cell plasticity model, cells bypass the lineage barrier and give rise to functionally and phenotypically different cells. For instance, transplanted bone marrow cells can differentiate into muscle cells^2^. Similarly, cells isolated from the brain and muscle are able to reconstitute the haematopoietic system^3^. The expression of lineage-specific transcription factors leads to the development of early progenitor cells that can give rise to differentiated cells^4^,^5^. Moreover, fibroblasts can be directly reprogrammed into committed differentiated cells^6^,^7^,^8^. Cellular dedifferentiation and transdifferentiation processes occur not only in these experimental settings but also during tissue regeneration. For example, on pancreatic tissue injury, pancreatic β cells are generated via self-duplication^9^ or transdifferentiation of α cells^10^. In addition, exocrine cells can be reprogrammed to become β cells^11^. Despite the biologic and pathologic significance of cell plasticity in tissue homeostasis and cancer, its underlying regulatory mechanism remains elusive.

Cancer SCs (CSCs) are regarded as a source of heterogeneous tumour cells and are responsible for tumour initiation, metastasis, recurrence and therapy resistance^12^,^13^. Although CSCs are somewhat similar to tissue SCs in that they are self-renewing, rare cell populations, their origin is still uncertain. Accumulating evidence indicates that CSCs originate from SCs, progenitor cells or differentiated cells^14^. However, these models have not been experimentally tested. It is possible that differentiated cells can be converted into progenitor cells or CSCs during tumorigenesis, similar to the cell dedifferentiation that has been observed in haematopoietic systems^4^,^5^. In this study, we found that the proliferating cell nuclear antigen-associated factor (*PAF*)-Wnt signalling axis converts normal epithelial cells into CSC-like (CSL) cells.

*PAF* (also known as *p15/KIAA0101/NS5ATP9/OEACT-1*) was initially identified as a proliferating cell nuclear antigen-interacting protein^15^. PAF modulates translesion DNA synthesis, a DNA bypass process, by facilitating the switch of the translesion DNA synthesis polymerase^16^. In recent times, we found that PAF, as a co-factor of β-catenin transcriptional complex, positively modulates Wnt signalling^17^. In this study, our results revealed that PAF is highly expressed in breast cancer cells and plays key roles in inducing mammary epithelial cell (MEC) plasticity and maintaining breast cancer cell stemness.

## Results

### Expression of PAF in breast cancer cells

**Figure 1:**
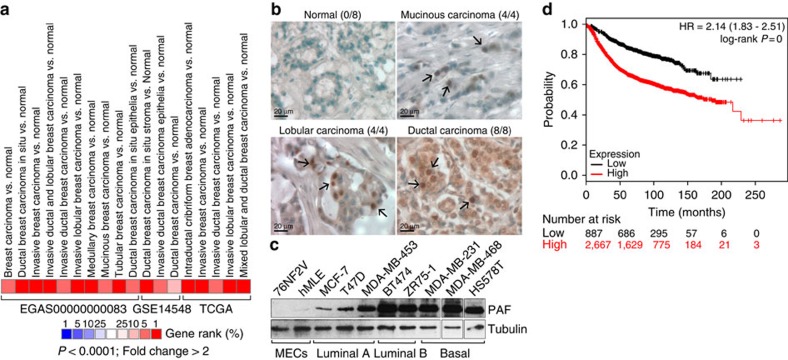
PAF expression in breast cancer cells. (**a**) *In silico* analysis of *PAF* expression in human breast cancer. Expression of *PAF* was analysed using publicly available Oncomine database (www.oncomine.org; *P*<0.0001; fold change>2; Student's *t*-test). (**b**) Expression of PAF in breast cancer. Breast cancer tissue microarray was immunostained for PAF using anti-PAF antibody and 3,3′-diaminobenzidine (DAB; brown). Haematoxylin was used for nuclear counterstaining (blue). Arrows: nuclear expression of PAF in breast cancer cells. Numbers in parentheses indicate number of PAF-positive samples out of number of total samples. Scale bars, 20 μm. (**c**) Expression of PAF in breast cancer cell lines. Human MECs and breast cancer cell lines were analysed for PAF expression using immunoblotting (IB) assays. (**d**) Survival graphs of breast cancer based on PAF expression. Using publicly available database (KM plotter; www.kmplot.com), relapse-free survival curves were analysed by PAF expression. A total of 3,554 patients; probe=202503_s_at; *P*=0. Probe 211713_x_at also showed the similar results (*P*=1.2 × 10^−5^). Log-rank *P*-values were calculated in kmplot database.

To identify the genes that play key roles in mammary tumorigenesis, we initially performed *in silico* analyses of publicly available breast cancer gene expression data sets (www.oncomine.org). We identified several genes that were highly expressed in breast cancer cells but not in normal breast tissues; *PAF* expression was remarkably upregulated in human breast cancer cells (Fig. 1a). To validate *PAF* upregulation in breast cancer, we performed immunohistochemical analyses using human breast cancer tissue microarrays. PAF expression was not detectable in normal MECs but was strongly expressed in the nuclei of invasive lobular, glandular and ductal adenocarcinoma cells (Fig. 1b). Consistently, PAF expression was significantly elevated in breast cancer cell lines but barely expressed in non-tumorigenic 76NF2V and hMLE human MECs (Fig. 1c). Of note is that luminal B and basal breast cancer cell lines show the higher expression of PAF, compared with luminal A breast cancer cell lines (Fig. 1c). In addition, a Kaplan–Meier analysis showed that high levels of PAF expression were strongly associated with poor prognosis in breast cancer (Fig. 1d and Supplementary Fig. 1). These results suggest that PAF expression is remarkably elevated in breast cancer cells.

### Acquisition of transforming activity by PAF

**Figure 2:**
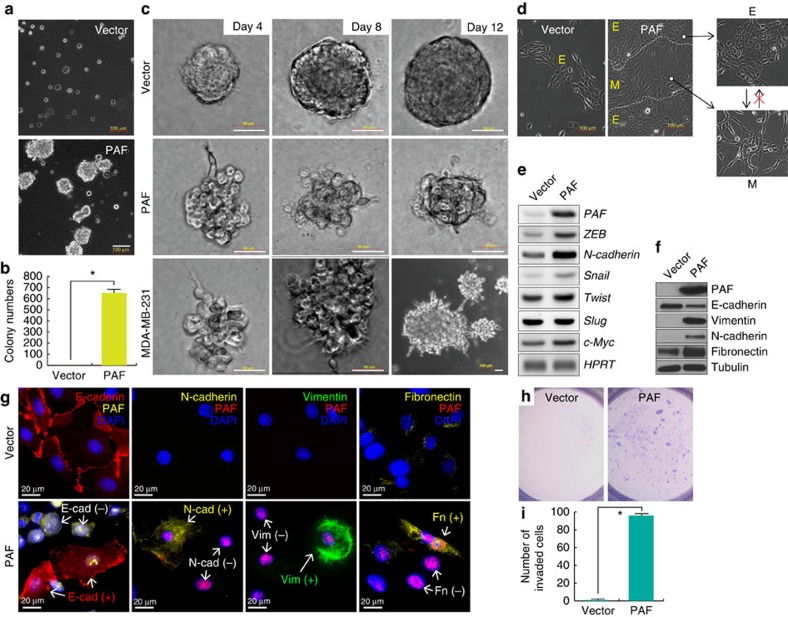
*In vitro* tumorigenic function of PAF. (**a**,**b**) Anchorage-independent growth of PAF-expressing MECs. Soft agar clonogenic assays were performed using 76NF2V-vector (control) or -PAF MECs. Each group of cells was grown in semisolid matrices (soft agar) for 3 weeks and analysed. (**a**) Phase-contrast images. (**b**) The number of colonies was quantified using colony counting. Student's *t*-test; error bars=s.e.m. Scale bars, 100 μm. (**c**) PAF-induced transformation of MECs. 76NF2V-vector, -PAF and MDA-MB-231 cells were cultured in three-dimensional Matrigel. Phase-contrast images of spheroid formation of each group of cells. MBA-MB-231 cells served as a positive control. Scale bars, 50 μm. (**d**) EMT by PAF. By light trypsinization, epithelial cell-like cells (**e**) and mesenchymal cell-like cells (**m**) were separated and cultured subsequently. It is noteworthy that e cells generate m cells but not *vice versa*. Scale bars, 100 μm. (**e**–**g**) Upregulation of EMT-related genes. 76NF2V cells (vector versus PAF) were analysed for reverse transcriptase–PCR (RT–PCR) (**e**), IB (**f**) and immunofluorescent staining (**g**). Scale bars, 20 μm. (**h**,**i**) Increased cell invasion by PAF. 76NF2V cells (vector versus PAF) were analysed for cell invasion assays. Images of cell migration (**h**); quantitative analysis (**i**). Student's *t*-test; error bars=s.e.m. *P<0.05; *N*>3.

Owing to the significant upregulation of *PAF* in breast cancer cells (Fig. 1), we hypothesized that PAF plays pro-tumorigenic roles in breast cancer. To test this, we assessed the effects of PAF expression on cellular transformation by evaluating the anchorage-independent growth of 76NF2V MECs, which do not express PAF (Fig. 1c). Similar to other MECs, 76NF2V-vector (control) cells did not grow in semisolid matrices. However, 76NF2V cells that stably expressed PAF (76NF2V-PAF) exhibited anchorage-independent growth (Fig. 2a,b). To further characterize the tumorigenic roles of PAF, we used a three-dimensional cell culture system. We plated an equal number (2,000 cells) of each group of cells (76NF2V-vector and -PAF) on Matrigel and cultured the cells for 12 days. 76NF2V-vector cells developed uniform round spheres. However, 76NF2V-PAF cells exhibited a loss of epithelial cell polarity and dendritic extension, as also shown in MDA-MB-231 breast cancer cells that served as a positive control (Fig. 2c).

Owing to the loss of epithelial cell polarity by PAF ectopic expression (see Fig. 2c), we next determined whether PAF expression induced epithelial–mesenchymal transition (EMT). Whereas 76NF2V-vector cells exhibited the typical epithelial cell morphological characteristics^18^, 76NF2V-PAF cells were heterogeneous (epithelial and mesenchymal) (Fig. 2d). To further characterize PAF-induced changes in cell morphological characteristics, we separated 76NF2V-PAF mesenchymal cells from epithelial cells by mild trypsinization. Each group of cells was evaluated according to their cellular morphological characteristics in the cell culture. Interestingly, 76NF2V-PAF epithelial cells continuously gave rise to both epithelial and mesenchymal cells, whereas mesenchymal cells generated only mesenchymal cells (Fig. 2d), accompanied by upregulated expression of mesenchymal markers and increased cell invasion (Fig. 2e–i). These results suggest that PAF plays tumorigenic roles, represented by anchorage-independent cell growth and EMT.

### Mammary ductal hyperplasia by PAF

**Figure 3:**
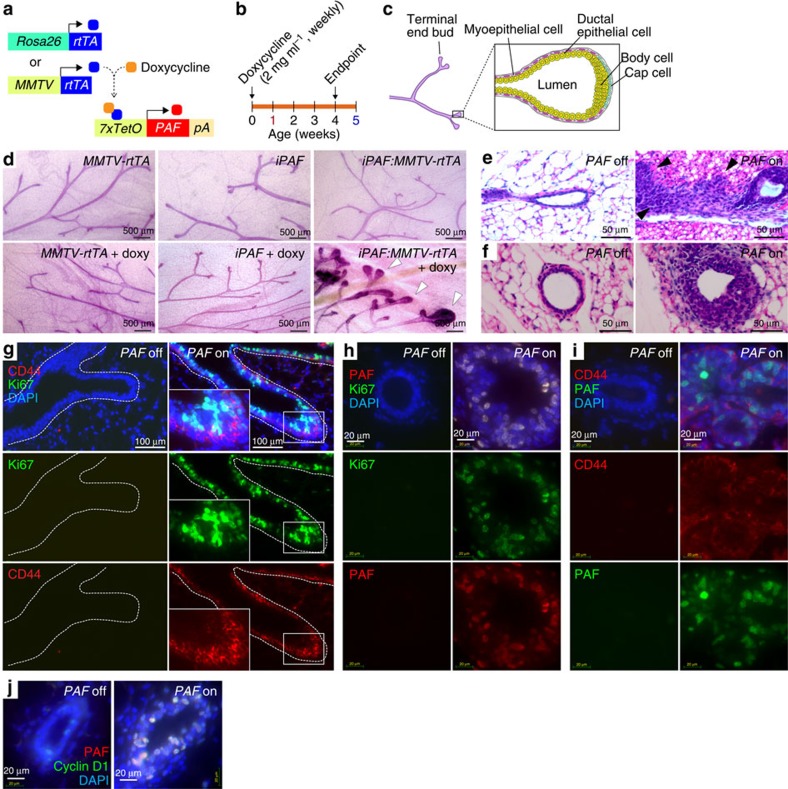
Mammary ductal hyperplasia by *PAF* conditional expression. (**a**) Schematic diagram of *PAF*-inducible mouse models. For MEC-specific induction of *PAF*, *MMTV-rtTA* driver was bred with *iPAF* strain. On doxycycline treatment in drinking water, activated rtTA binds to TetO and transactivates *PAF* expression in MECs. Alternatively, *Rosa26-rtTA* strain was used. (**b**) Doxycycline administration strategies. Doxycycline was administrated in drinking water (5% sucrose) for each time point. (**c**) Illustration of mammary duct structure. (**d**–**f**) Ductal hyperplasia by PAF conditional expression. *MMTV-rtTA:iPAF* (experimental group) or *MMTV-rtTA* (control) mice were treated with doxycycline (2 mg ml^−1^) for 30 days after weaning. Longitudinal (**d**) or transverse (**e**) sectioned mammary glands were stained with haematoxylin and eosin. Arrowheads (white): hyperplastic TEBs; arrowheads (black): infiltrated ductal epithelial cells (**f**). Hyperproliferation of ductal epithelial cells by PAF. Ki67 immunostaining of longitudinal section. Scale bars, 500 μm (**d**); 50 μm (**e**,**f**). (**g**–**i**) Upregulation of CD44 by conditional expression of PAF. Ki67 (green) and CD44 (red) immunostaining of mammary ducts. To exclude indirect effects of PAF expression on cell proliferation, doxycycline was administrated for 7 days instead of 30 days. Scale bars, 100 μm (**g**); 20 μm (**h**,**i**). (**j**) Upregulation of Cyclin D1 by conditional expression of PAF. Doxycycline treated for 7 days. Scale bars, 20 μm.

Next, we determined the physiologic roles of PAF *in vivo* by mimicking a condition of upregulated PAF in breast cancer. We used genetically engineered mouse models with a tetracycline-inducible gene expression system, as described previously^17^,^19^. For conditional expression of PAF, a *PAF*-inducible strain (*iPAF*) was bred with *MMTV-rtTA* or *Rosa26-rtTA* strains. Next, *MMTV-rtTA:iPAF* or *Rosa26-rtTA:iPAF* compound mice were treated with doxycycline, an analogue of tetracycline, which leads to the induction of *PAF* expression (Fig. 3a). After 4 weeks of doxycycline treatment, we assessed the development of the mammary ducts of the *iPAF*, *MMTV-rtTA*, *iPAF:MMTV-rtTA*, *iPAF*+doxycycline and *MMTV-rtTA*+doxycycline strains (control groups), and the *MMTV-rtTA*:*iPAF*+doxycycline compound strain (experimental group) (Fig. 3b). During development, the mammary ducts elongate and bifurcate, which is mainly driven by the activation of mammary SCs located in the terminal end buds (TEBs) (Fig. 3c). Surprisingly, mice that conditionally expressed *PAF* displayed ductal hyperplasia, which manifested with enlarged TEBs, increased ductal bifurcation, side branching and thickened ducts (Fig. 3d). A microscopic analysis further showed that the mammary ductal epithelium was remarkably hyperplastic in *MMTV-rtTA:iPAF* mice, whereas the *iPAF* only (control) mice displayed a single layer of ductal epithelium (Figs 3e,f). Similarly, *Rosa26-rtTA* driver-induced *PAF* expression also induces mammary ductal hyperplasia (Supplementary Fig. 2).

To exclude any indirect effects of the long-term induction of *PAF*, we also examined the mammary tissues of mice treated with doxycycline for 7 days. The short-term administration (7 days) of doxycycline was sufficient to induce hyperproliferation of ductal epithelial cells, as displayed by Ki67-positive cells in both luminal and basal epithelial (BE) cells. However, the mammary tissues of control mice exhibited Ki67-positive cells only in TEBs (Fig. 3g,h). Previously, we found that PAF hyperactivates Wnt signalling as a co-factor of the β-catenin transcription complex^17^. Thus, we determined whether *PAF* conditional expression activates Wnt signalling by assessing the expression of *CD44*, a β-catenin target gene^20^. We found that CD44 was strongly upregulated in the entire mammary epithelium of PAF-induced mice (doxycycline for 7 days) but was weakly expressed in the TEBs of control mice (Fig. 3g,i). Additional β-catenin target gene, *Cyclin D1*, was significantly upregulated by PAF conditional expression (Fig. 3j). Consistently, we observed that conditional expression of PAF upregulates β-catenin protein (7 days of doxycycline treatment) and induces the nuclear translocation of β-catenin (30 days of doxycycline) (Supplementary Fig. 3). These results suggest that the conditional expression of PAF induces mammary ductal hyperplasia with activation of Wnt signalling.

We induced PAF expression in mammary epithelium for a long-term analysis. The conditional expression of *PAF* for up to 8 months did not result in a mammary tumour (data not shown), suggesting that PAF *per se* is not sufficient to initiate mammary tumorigenesis. Next, we determined whether additional factors (inactivation of p53 or pRb) are required for PAF-induced tumour development. We performed xenograft transplantation assays using various 76NF2V MECs expressing E6, E7 or TERT. We subcutaneously injected MECs that stably expressed PAF into nude mice. 76NF2V and HMLE did not form tumours *ex vivo*^21^. However, 76NE6 or 76NE6/TERT cells that stably expressed PAF developed the tumours in immunocompromised mice (Supplementary Fig. 4). Of note, p53 was inactivated by E6 protein in 76NE6 and 76NE6/TERT MECs. Thus, these results indicate that in the setting of p53 inactivation, PAF contributes to mammary tumour initiation. Our results suggest that the conditional expression of PAF induces mammary ductal hyperplasia and initiates tumour development in the setting of *p53* inactivation.

### Generation of cancer SC-like cells by PAF-Wnt axis

**Figure 4:**
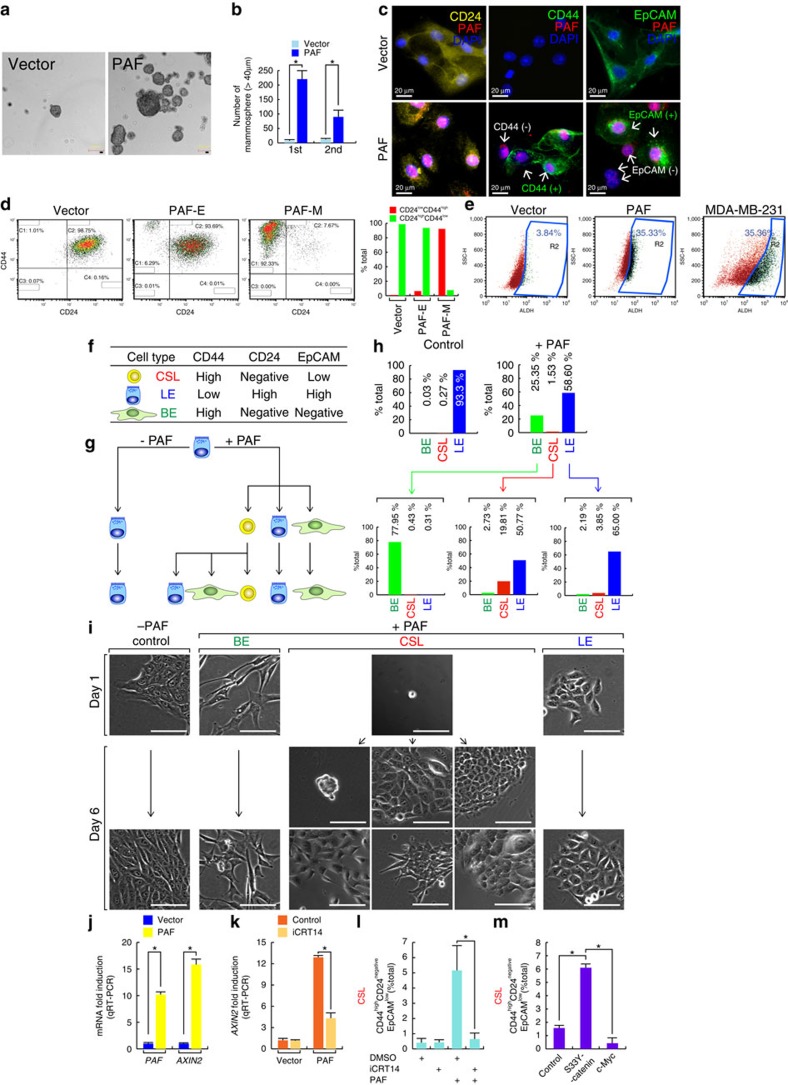
Generation of CSC cells by PAF-Wnt signalling axis. (**a**,**b**) Increased mammosphere formation by PAF. 76NF2V-GFP or -PAF cells were cultured at low density (500 cells per ml in 6-well plate) in suspension condition. Primary mammosphere (at 10 days) were dissociated and re-plated for multiple propagations (12 days). (**a**) Phase-contrast images. (**b**) The number of mammospheres was quantified using plate counter. Scale bar, 100 μm. (**c**) Expression of breast CSC markers in PAF-expressing MECs. 76NF2V-vector and -PAF cells were analysed for immunostaining with each antibody. Scale bars, 20 μm. (**d**) CD24 downregulation and CD44 upregulation by PAF ectopic expression. 76NF2V-vector and -PAF cells were analysed using FACS for CD24 and CD44 cell surface markers. PAF expression converted MEC properties from CD24^high^CD44^low^ to CD24^low^CD44^high^ in 76NF2V-PAF epithelial cell-like cells (**e**) and CD24^low^CD44^high^ in 76NF2V-PAF mesenchymal-like cells (**m**). The representative results were shown (*N*=3). (**e**) Increased ALDH activity by PAF. 76NF2V-vector and -PAF cells were analysed for ALDH activity using FACS. MDA-MB-231 served as a positive control. The representative results were shown (*N*=3). (**f**,**g**) Schematic diagram of PAF-induced cell plasticity. (**f**) Based on expression of three cell surface markers, CD44, CD24 and EpCAM, 76NF2V-control and -PAF cells can be sorted using FACS. (**g**) In hypothetical model, PAF expression converts MECs into SC-like cells that generate heterogeneous cell population. BE, basal epithelial cells; CSL, cancer SC-like cells; LE, luminal epithelial cells. (**h**,**i**) PAF-induced cell heterogeneity. Each group of cells (CSL, LE and BE) from 76NF2V-PAF cells was sorted using FACS and cultured for 6 days. Next, cells were reanalysed for cell properties using FACS (CD24, CD44 and EpCAM). The representative results were shown (*N*=3) (**h**). Phase-contrast images (**i**). Scale bars, 50 μm. (**j**) Upregulation of *AXIN2* by PAF. 76NF2V-control and -PAF cells were analysed for *AXIN2* expression using quantitative reverse transcriptase–PCR (qRT–PCR). *AXIN2* fold induction was normalized by *GAPDH* expression. Student's *t*-test; error bars=s.e.m. (**k**) Suppression of PAF-induced *AXIN2* upregulation. 76NF2V-control (vector) and -PAF cells were treated with iCRT14 (100 μM for 24 h) and analysed for *AXIN2* expression. Student's *t*-test; error bars=s.e.m. (**l**) Suppression of PAF-induced generation of breast CSL cells by β-catenin inhibition. 76NF2V-control and -PAF cells were treated with iCRT14 (100 μM for 24 h) and analysed using FACS for SC population (CD44^high^CD24^negative^EpCAM^low^). Dimethyl sulfoxide (DMSO) vehicle served as a negative control for iCRT14. Student's *t*-test; error bars=s.e.m. (**m**) Generation of CSL cells by active β-catenin. 76NF2V MECs stably expressing S33Y-β-catenin or c-Myc were analysed for CD24, CD44 and EpCAM expression using FACS. Student's *t*-test; error bars=s.e.m. *P<0.05; *N*<3.

CSCs are a rare cell population that replenishes the entire tumour; they also play essential roles in tumorigenesis and metastasis^12^,^13^. Our results show that (a) PAF is specifically expressed in breast cancer cells (Fig. 1b) and (b) PAF expression induces the cellular transformation of MECs (Fig. 2a–c) with Wnt signalling activation (Fig. 3g). In addition, the results of our previous study showed that PAF expression is associated with cell stemness in embryonic and intestinal SCs^17^. These results led us to hypothesize that PAF expression is associated with the development or maintenance of CSCs. Therefore, to assess the impact of PAF on the self-renewal capacity of MECs, we performed mammosphere assays. 76NF2V-PAF cells formed remarkable mammospheres, whereas 76NF2V-vector control cells did not develop mammospheres (Fig. 4a). In addition, only 76NF2V-PAF cells formed the successive mammospheres in the secondary and tertiary cultures by single-cell dissociation (Fig. 4b). Similarly, hMLE stably expressing PAF displays the increase in mammosphere formation (Supplementary Fig. 5).

Next, we examined the expression of breast CSC-associated markers in 76NF2V cells. Immunofluorescence (IF) staining showed that CD44 was upregulated in 76NF2V-PAF, while the expression of EpCAM and CD24 was heterogeneous and slightly downregulated by PAF expression (Fig. 4c). To better analyse the expression of breast CSC markers (CD24^low^ and CD44^high^)^22^, we used fluorescence-activated cell sorting (FACS). 76NF2V-vector cells exhibited CD24^high^ and CD44^low^ expression, indicating their luminal epithelial (LE) properties^23^. However, 76NF2V-PAF cells displayed CD24^low^ and CD44^high^ expression (Fig. 4d). Aldehyde dehydrogenase (ALDH), another breast CSC marker^24^, was significantly upregulated in 76NF2V-PAF cells, similar to MDA-MB-231, which served as a positive control (Fig. 4e). These results suggest that PAF expression converts MECs into breast CSL cells.

Having found that PAF expression upregulates breast CSC markers (see Fig. 4c–e), we hypothesized that it induces the cell plasticity that converts MECs into breast CSL cells. We first determined whether PAF-expressing MECs generate cellular heterogeneity. On the basis of the differential expression of CD24, CD44 and EpCAM cell surface markers^25^, we sorted MECs into LE cells (CD44^low^CD24^high^EpCAM^high^), BE cells (CD44^high^CD24^negative^EpCAM^negative^) and breast CSL cells (CD44^high^CD24^negative^EpCAM^low^) (Fig. 4f,g). 76NF2V-vector control cells exhibited LE characteristics (93%), confirming their luminal origin^23^. However, 76NF2V-PAF cells gave rise to LE, BE and CSL cells (Fig. 4h). Next, we determined whether the CSL cells generated by PAF successively give rise to cellular heterogeneity. We sorted 76NF2V-PAF cells into LE, BE and CSL cells using FACS and cultured each group of cells for 6 days. We then reanalysed each cell population on the basis of CD24, CD44 and EpCAM expression. The sorted BE and LE cells reproduced BE and LE, respectively. Of note, LE cells also partially generated some BE cells (Fig. 4h and Supplementary Fig. 6), which recapitulates EMT induction by PAF (Fig. 2d-i). Interestingly, we observed that CSL cells gave rise to LE, BE and CSL cell populations (Fig. 4h and Supplementary Fig. 6). The phase-contrast images also showed that the sorted CLS cells generated various cells with different morphological characteristics after 6 days of culture (Fig. 4i), consistent with IF staining results showing PAF-induced heterogeneous expression of CD44, EpCAM and EMT-related genes (Figs 2g and 4c). These results suggest that CSL cells induced by PAF generates cellular heterogeneity. In addition, owing to differential capability of PAF expression in generating CSCs, we asked whether the different level of PAF expression determines each cell fate, by assessing PAF expression. However, we found that there is no significant difference in *PAF* transcription among those three cell populations (Supplementary Fig. 7), implying that posttranslational modification of PAF might be involved in modulating PAF activity.

Given that PAF activates the β-catenin transcriptional complex and promotes Wnt target gene transcription^17^, we determined whether it generates CSL cells via Wnt/β-catenin signalling. Ectopic expression of PAF remarkably upregulated *AXIN2*, a well-established β-catenin target gene^26^, in 76NF2V-PAF cells (Fig. 4j), which was suppressed by iCRT14 treatment (Fig. 4k), recapitulating that PAF activates Wnt/β-catenin signalling in mammary epithelium (see Fig. 3g). We next determined whether PAF-induced CSL cell generation is inhibited by iCRT14, an inhibitor of β-catenin–T-cell factor (TCF) binding^27^. iCRT14-treated 76NF2V-PAF cells did not generate a CSL cell population (Fig. 4l). Conversely, ectopic expression of S33Y-β-catenin, a constitutively active form of β-catenin^28^, generated CSL cells (Fig. 4m), which was also suppressed by iCRT14 (Supplementary Fig. 8). It was previously shown that ectopic expression of *c-Myc*, a direct target of β-catenin^29^, reprogrammes epithelial and cancer cells into SC-like cells^30^; this prompted us to determine whether PAF-induced cell plasticity is due to β-catenin-mediated *c-Myc* transactivation. We found that ectopic expression of c-Myc *per se* did not give rise to CSL cells from 76NF2V MECs. Instead, it induced the overall EMT (Supplementary Fig. 9), different from a previous study that suggested that EMT can give rise to breast CSL cells^31^. These results suggest that the PAF-Wnt/β-catenin signalling axis converts MECs into breast CSL cells.

### *PAF* transactivation by stemness-associated factors

**Figure 5:**
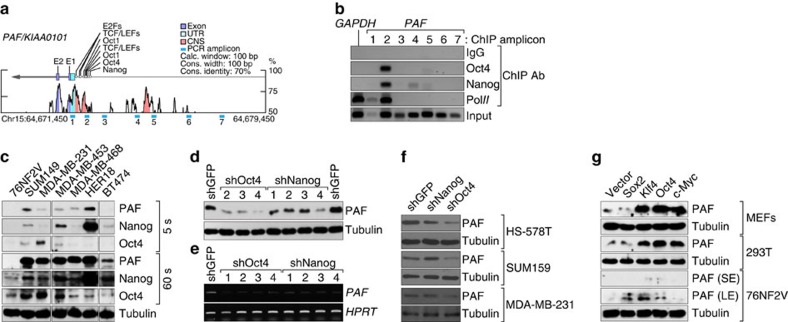
*PAF* transactivation by Oct4 and Nanog. (**a**) *PAF* promoter analysis using VISTA genome browser. CNS, conserved non-coding sequence; TBE, TCF-binding elements; UTR, untranslated region. Green bars, PCR amplicons (1∼7). (**b**) Oct4 and Nanog occupy *PAF*'s proximal promoter. ChIP analysis of HER18. GAPDH promoter, a negative control for occupancy of Oct4 and Nanog; IgG, a negative control for IP; Input, 10% were used for ChIP–PCR; RNA polymerase II (Pol II), a positive control for ChIP. (**c**) Expression analysis of PAF, Oct4 and Nanog. IB assays. LE, long exposure; SE, short exposure. (**d**–**f**) Depletion of either Oct4 or Nanog downregulates *PAF* expression. HER18 cells stably expressing short hairpin RNAs (shRNAs) were analysed for IB (**d**) and semi-quantitative RT–PCR (**e**). Other breast cancer cell lines were stably transduced with lentiviruses encoding shRNAs against Oct4 or Nanog. PAF expression was analysed by IB. (**g**) Ectopic expression of stemness-associated factors upregulates *PAF* expression. Each cell line was transfected with indicated plasmids. Twenty-four hours after transfection, cells were collected for IB analysis. MEFs, mouse embryonic fibroblasts.

Next, we determined how *PAF* expression is upregulated in breast cancer cells. We analysed the promoter sequence of *PAF* to identify its transactivating transcription factors. Using MatInspector software (genomatix.de), we identified several transcription factors that may bind to *PAF* promoter (Supplementary Table 1). Interestingly, we found several consensus binding sites for Oct1, Oct4, Nanog and TCF/lymphoid enhancer-binding factor 1 (LEFs) at *PAF*'s proximal promoter (Fig. 5a). Oct4, Sox2, Nanog and TCFs function as master regulators of stemness in embryonic SCs^32^,^33^ and their ectopic expression reprogrammes differentiated cells into a pluripotent SC lineage^34^. The results of recent studies also suggest that these stemness-associated factors are highly expressed in breast cancer and CSCs^35^,^36^,^37^. This evidence led us to determine whether Oct4 and Nanog transactivate *PAF*. Indeed, chromatin immunoprecipitation (ChIP) assays showed that Oct4 and Nanog occupied *PAF*'s proximal promoter in HER18 cells (Fig. 5b). We also found that PAF, Oct4 and Nanog were upregulated in breast cancer cell lines (Fig. 5c). Next, we determined whether Oct4 and Nanog are required for *PAF* expression. Indeed, depletion of either endogenous Oct4 or Nanog downregulated PAF expression in HER18 cells (Fig. 5d,e) and other breast cancer cell lines (Fig. 5f). Conversely, ectopic expression of these stemness factors induced PAF expression in mouse embryonic fibroblasts and 293T cells, and induced slight expression in 76NF2V cells (Fig. 5g). These results suggest that Oct4 and Nanog transactivate *PAF* in breast cancer cells.

### PAF is required for maintaining breast cancer cell stemness

**Figure 6:**
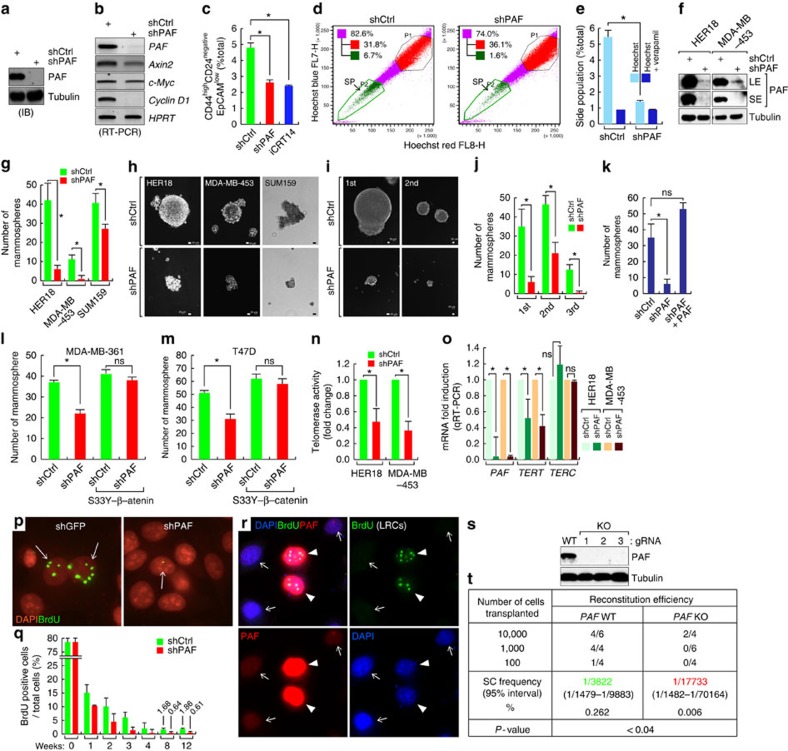
PAF is required for breast CSC maintenance. (**a**,**b**) Depletion of endogenous PAF by shPAF. HER18 human breast cancer cells were transduced with lentivirus encoding shCtrl (control) or shPAF (PAF depletion) and selected with puromycin. PAF knockdown was confirmed using IB (**a**) and semi-quantitative reverse transcriptase–PCR (RT–PCR) (**b**). Tubulin and *HPRT* served as internal controls. (**c**–**e**) PAF depletion decreases breast CSC population. HER18 cells (shCtrl (control), shPAF or iCRT14 treated) were analysed by flow cytometry and CSL population (CD44^high^CD24^negative^EpCAM^low^) was quantified (**c**). Side population analysis (**d**). Verapamil-treated samples served as negative controls (**e**). Student's *t*-test; error bars=s.e.m. (**f**) Depletion of endogenous PAF by short hairpin RNAs (shRNAs) in breast cancer cell lines. IB analysis. (**g**–**j**) Decrease in mammosphere formation by PAF depletion. Quantification of the number of mammospheres (**g**) and phase-contrast images (**h**). Sequential mammosphere formation of HER18 cells (**i**,**j**). Of note, SUM159 and MDA-MB-453 cells did not form clear mammospheres. Student's *t*-test; error bars=s.e.m. Scale bars, 20 μm. (**k**) Ectopic expression of PAF rescues inhibition of mammosphere formation by PAF depletion. HER18 cells were stably transduced by PAF and analysed for mammosphere formation. Student's *t*-test; error bars=s.e.m. (**l**,**m**) β-Catenin rescues PAF depletion-induced inhibition of mammosphere formation. shCtrl- and shPAF-transduced breast cancer cell lines were stably transfected with S33Y-β-catenin and assessed for mammosphere formation. Student's *t*-test; error bars=s.e.m. (**n**) Decreased telomerase activity by PAF depletion. Cells (shCtrl versus shPAF) were analysed for telomerase activity using TRAPEZE RT kits. Student's *t*-test; error bars=s.e.m. (**o**) Downregulation of *TERT* by PAF depletion. Student's *t*-test; error bars, s.e.m. Quantitative reverse transcriptase–PCR (RT–PCR) analysis. Of note, *TERC* was not changed by PAF depletion. (**p**,**q**) Decrease in LRCs in PAF-depleted HER18 cells. HER18 cells were labelled with 5-bromodeoxyuridine (BrdU) for 24 h and analysed for LRCs by counting BrdU-positive cells (*n*, microscopic images; *o*, graph). Of note, initially, equal number of cells (shCtrl versus shPAF) displayed BrdU incorporation. Student's *t*-test; error bars=s.e.m. (**r**) High expression of PAF in LRCs. HER18 LRCs (2 months) were analysed for IF staining. Arrowheads: PAF^high^ and BrdU^positive^ cells; arrows: PAF^low^ and BrdU^negative^ cells. (**s**) Somatic KO of *PAF* by CRISPR. Wesrern blot (WB) analysis of MDA-MB-231 cells. (**t**) *PAF* KO inhibits stemness of breast cancer cells. MDA-MB-231 (*PAF* WT and KO) cells were transplanted into the cleared fat pad of immunocompromised mice. Twelve weeks after transplantation, tumour formation was assessed by extreme limiting dilution analysis. *P<0.05; NS, not significant (*P*⩾0.05); *N>*3.

Given the PAF-induced conversion of MECs into CSL cells (see Fig. 4), we hypothesized that PAF is required for the maintenance of breast cancer cell stemness. It was previously shown that human breast cancer cell lines contain CSL cells (∼5% of total cells), as assessed by CD24, CD44 and EpCAM expression^13^,^25^,^38^. Because of the high expression level of PAF in breast cancer cells (Fig. 1), we used loss-of-function approaches using lentiviruses that encode short hairpin RNA against green fluorescent protein (GFP) (control; shGFP) or PAF (shPAF) (Fig. 6a,b). We found that PAF depletion downregulated β-catenin target genes (*AXIN2*, *c-Myc* and *Cyclin D1*) in breast cancer cells, recapitulating that PAF activates Wnt/β-catenin signalling^17^ (Figs 3g and 4j). Next, we assessed the effects of PAF depletion on the quantity of CSL cells (CD44^high^CD24^negative^EpCAM^low^). Intriguingly, we found that PAF depletion or iCRT14 (inhibition of β-catenin-mediated gene transactivation) decreased the number of breast CSCs (Fig. 6c). Similarly, side population assays also showed that PAF depletion diminished the CSC population (Fig. 6d,e). In addition, iCRT14 treatment inhibits mammosphere formation of breast cancer cells (Supplementary Fig. 10). It is noteworthy that PAF knockdown and iCRT14 treatment did not affect breast cancer cell proliferation (data not shown). In addition, PAF depletion inhibited mammosphere formation in breast cancer cell lines (SUM159, HER18, MDA-MB-435 and MDA-MB-453) (Fig. 6f–h). Subsequent dissociating and replating of cells from the primary mammospheres showed a similar inhibition of mammosphere formation in PAF-depleted cells (Fig. 6i,j), which was rescued by ectopic expression of PAF (Fig. 6k). Furthermore, we found that ectopic expression of S33Y-β-catenin rescues PAF depletion-induced inhibition of mammosphere formation (Fig. 6l,m). These results suggest that PAF-induced stemness maintenance is mediated by Wnt/β-catenin signalling.

Next, we assessed the effects of PAF depletion on telomerase, a specific marker for self-renewing cells^39^. PAF depletion decreased telomerase activity (Fig. 6n), with downregulation of *TERT*, a catalytic subunit of telomerase, but not *TERC*, an RNA template of telomerase (Fig. 6o). Moreover, label-retaining cell (LRC) assays^40^ showed that PAF depletion decreased the LRC population, which is considered the SC population (Fig. 6p,q). We also determined whether expression of PAF is enriched in the LRCs of HER18 breast cancer cells. Intriguingly, we found that PAF was relatively highly expressed in LRCs compared with in non-LRCs (Fig. 6r). These results suggest that PAF expression is upregulated in breast CSCs. Next, to determine the effects of *PAF* knockout (KO) on *in vivo* tumour formation, we generated *PAF* KO MDA-MB-231 cell lines using the clustered regularly interspaced short palindromic repeat (CRISPR) gene targeting approach (Fig. 6s). *PAF* wild-type and KO cells were transplanted into the cleared mammary fat pads at a limiting dilution. Twelve weeks after transplantation, transplants with *PAF* KO cells had fewer outgrowths than did control cells, with a significant decrease in the frequency of mammary gland-reconstituting cells of *PAF* KO cells (0.023 fold) (Fig. 6t). These results suggest that the PAF-Wnt signalling axis is required for the maintenance of breast cancer cell stemness.

### Identification of clinical cancer drugs that target PAF

**Figure 7:**
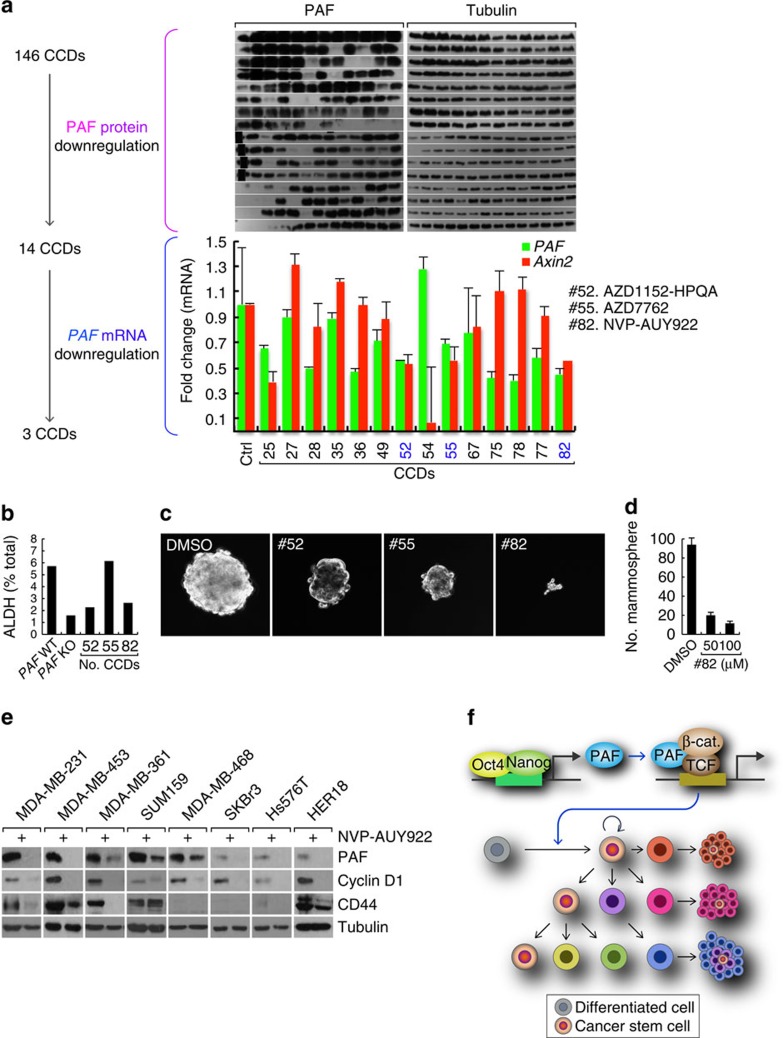
Identification of clinical cancer drugs targeting PAF. (**a**) Total 146 CCDs were treated in HER18 cells (1 μM; 24 h) for screening potential PAF inhibitors. Fourteen CCDs downregulating PAF protein are identified by IB assays. Next, among 14 CCDs, 3 CCDs (blue) were selected by their inhibitory effects on *PAF* and *AXIN2* transcription using quantitative reverse transcriptase–PCR (qRT–PCR). Error bars=s.e.m. (**b**) Decreased ALDH activity by CCDs. HER18 cells were treated with three CCDs (100 nM; 24 h) and analysed for ALDH activity using FACS. HER18 *PAF* KO cells served as a negative control. Diethylaminobenzaldehyde (DEAB)-treated cells were also tested as a negative control (data not shown). (**c**,**d**) Decrease in mammosphere formation by NVP-AUY922. Phase-contrast images (**c**) and quantification of the number of mammosphere (**d**). Error bars=s.e.m. (**e**) Downregulation of β-catenin target genes by NVP-AUY922. Breast cancer cell lines were treated with NVP-AUY922 (100 nM, 36 h) and analysed by IB assays. (**f**) Illustration of working model: PAF-Wnt signalling axis in breast cancer cell stemness.

Because of specific expression of PAF in breast cancer cells, but not in the mammary gland (see Fig. 1), targeting PAF might specifically target breast cancer cells that express PAF. To this end, we screened 146 clinical cancer drugs (CCDs) to determine their effects on PAF expression (Supplementary Table 2). First, we treated MDA-MB-231 cells with 146 CCDs for 24 h and determined their effects on the PAF protein level. We identified 14 CCDs that significantly downregulate PAF protein. For the second round of screening, we treated HER18 cells with 14 selected CCDs and analysed the transcription level of *PAF* and *AXIN2* by quantitative reverse transcriptase–PCR. Finally, we chose three CCDs (AZD1152-HPQA (52; aurora B kinase inhibitor), AZD7762 (55; Chk1 inhibitor) and NVP-AUY922 (82; Hsp90 inhibitor)) on the basis of their effects on the downregulation of both *PAF* and *AXIN2* expression (Fig. 7a). Next, we determined the effects of these three CCDs on the stemness of breast cancer cells by assessing ALDH activity. We found that treatment with AZD1152-HPQA and NVP-AUY922 significantly reduced ALDH activity, as shown in *PAF* KO cells (Fig. 7b). Subsequent mammosphere assays showed that NVP-AUY922 remarkably inhibits mammosphere formation (Fig. 7c,d). In addition, NVP-AUY922 downregulates β-catenin target genes in breast cancer cell lines (Fig. 7e). These results suggest that NVP-AUY922 is a CCD that reduces breast cancer cell stemness by molecular targeting of PAF.

## Discussion

We previously found that PAF is highly expressed in colorectal and pancreatic cancer, and hyperactivates the Wnt/β-catenin and mitogen-activated protein kinase signalling pathways, which results in the initiation of tumorigenesis in mouse models^17^,^41^. However, we found that PAF depletion or ectopic expression does not affect mitogen-activated protein kinase signalling activity in MECs and breast cancer cell lines (Supplementary Fig. 11). In addition, despite the role of PAF in DNA repair, PAF does not affect DNA damage response (Supplementary Fig. 12). These results suggest that PAF plays the distinct roles in breast cancer, unlike other cancer. Interestingly, PAF is not expressed in terminally differentiated cells but is specifically expressed in embryonic stem and intestinal SCs^17^, implying that it has roles in cell stemness. Interestingly, *in silico* analyses showed that PAF is also highly expressed in breast cancer cells (see Fig. 1), which led us to study its potential tumorigenic roles in breast cancer. Herein, we found that PAF induces the conversion of MECs into CSL cells via Wnt/β-catenin signalling. In contrast, PAF depletion leads to the loss of stemness of breast cancer cells, which is required for the maintenance of breast cancer cell stemness (Fig. 7f). Furthermore, our drug-repurposing approaches identified the CCDs inhibiting breast cancer cell stemness by targeting PAF.

Deregulation of Wnt signalling is associated with various human cancers^42^,^43^. In a mouse model, *MMTV*-driven ectopic *Wnt-1* expression induced mammary hyperplasia and adenoma^44^. However, the tumorigenic roles of Wnt signalling in human breast cancer have been controversial^45^,^46^. In recent times, it was suggested that Wnt/β-catenin signalling deregulation is associated with triple-negative breast cancer^47^,^48^. Of note, unlike colorectal cancer, breast cancer cells exhibit infrequent genetic mutations of *APC* or *β-catenin/CTNNB1* (refs 49, 50), which implies that additional regulatory mechanisms of Wnt signalling exist. Hence, it is plausible that PAF overexpression is one of the regulatory mechanisms of Wnt signalling hyperactivation in breast cancer, including triple-negative breast cancer.

The traditional study of Wnt signalling in tumorigenesis has mainly focused on the effects of Wnt signalling hyperactivation on cell proliferation, which are mainly due to β-catenin-mediated transcriptional activation of *c-Myc*^29^ and *Cyclin D1* (ref. 51), which promotes cell proliferation. Despite the crucial roles of Wnt signalling in cell specification during embryogenesis, how cell fate is determined in tumorigenesis is poorly understood. It was shown that Wnt signalling increases the number of mammary SCs and progenitor cells^52^,^53^. In addition, Wnt signalling is required for the maintenance of mammary SCs^54^ and Wnt/β-catenin signalling activation leads to the development of leukaemia SCs^55^. The results of these studies suggest that Wnt signalling hyperactivation enriches CSL cells. Intriguingly, *MMTV-Wnt1*- or -Δβ-catenin-derived mammary tumours contain both myo- and LE cell lineages, unlike other breast cancer mouse models, including *MMTV-Neu*, *MMTV-PyMT* and *MMTV-H-Ras*^52^,^56^. In addition, it was shown that Wnt-responsive cells constitute various types of MECs during mammary development^57^. Thus, Wnt signalling might be associated with the generation of tumour cell heterogeneity. Nonetheless, its detailed molecular mechanisms remain ambiguous. Our findings reveal that the PAF-Wnt signalling axis converts MECs into CSL cells, which further develop cell heterogeneity. This may explain the molecular mechanism of the Wnt signalling-induced generation of tumour cell heterogeneity.

In line with our findings, accumulating evidence suggests that various cells transform into CSL cells by hypoxia^58^, EMT^31^ or inflammation^59^. Spontaneous cell plasticity towards CSL cells was also observed^60^. Although *c-Myc*, a direct transcriptional target of β-catenin, was shown to trigger self-renewal reprogramming^30^, we observed that it induces EMT but not the generation of CSL cells (see Fig. 4l). EMT is the conversion of cell fate from epithelial cells to mesenchymal cells. Despite upregulation of EMT-associated genes and cell morphological change by PAF ectopic expression in MECs, we found that, unlike EMT, PAF induces the partial EMT, indicated by IF staining (see Fig. 2) and FACS analysis (see Fig. 4). This is also represented by the difference in CSL cell generation between c-Myc and PAF. Although c-Myc induces the complete conversion of LE to BE (Supplementary Fig. 9), PAF expression generates cellular heterogeneity (LE, BE and CSL), which supports our model that PAF-induced cell plasticity generates CSL rather than EMT. These results suggest that other Wnt/β-catenin target genes besides *c-Myc* are associated with PAF-induced cell plasticity. Thus, identifying the key downstream target genes that initiate cell plasticity will be of great interest, which will elucidate the transcriptional circuits of cancer cell stemness.

Given that PAF is highly upregulated in breast cancer cells, it is likely to be that PAF-induced cell plasticity is a key pathologic process in maintaining breast CSCs. Intriguingly, *PAF* ablation inhibits the stemness of breast cancer cells, which suggests that reversing cell plasticity by molecular targeting of PAF could be translated into a therapeutic intervention for breast cancer. To further develop methods for targeting PAF, we screened 146 CCDs on the basis of their effects on PAF expression. We found that AZD1152, AZD7762 and NVP-AUY922 markedly downregulated both protein and msessenger RNA levels of PAF and significantly reduced breast cancer cell stemness. AZD1152, an aurora B kinase inhibitor^61^, induces radiation response in p53-deficient cancer cell including colorectal and pancreatic cancer cells^62^,^63^. AZD7762 enhances the activity of DNA-damage reagents in a p53-dependent manner^64^. In xenograft model, in combination with DNA damage reagents, AZD7762 increases cytotoxicity and inhibits tumour growth^65^,^66^. It was previously shown that NVP-AUY922 exhibits antitumour activity in breast cancer cells (Supplementary Fig. 13)^67^. Moreover, given the specific expression of PAF in the subset of cancer cells, but not in normal cells, targeting the PAF molecule may minimize its harmful effects on normal tissues. Of note, *PAF* KO mice are also viable^68^, which further supports the benefit of molecular targeting of PAF. Thus, our results suggest that NVP-AUY922's effects on PAF downregulation can be translated into the development of targeted therapy against cancer cell stemness. Collectively, our findings reveal a previously unsuspected role of the PAF-Wnt signalling axis in maintaining cancer cell stemness by controlling cell plasticity.

## Methods

### Kaplan–Meier analysis

Kaplan–Meier analysis was performed using publicly available database (kmplot.com)^69^ with the following options: relapse-free survival; splitting patients by lower quartile split, judging from the numbers at risk at time; two probes for *PAF/KIAA0101*=202503_s_at and 211713_x_at; and without restricting analysis to subtypes.

### Mammalian cell culture

Cell lines (MCF-7, T47D, MDA-MB-453, BT474, ZR75-1, MDA-MB-231, MDA-MB-468, HER18, SUM149, SUM159, 293T and HS578T) were purchased from American Type Culture Collection (ATCC) and maintained in DMEM medium containing 10% fetal bovine serum. 76NF2V and hMLE MEC cells, generous gifts from Drs Vimla Band and Li Ma, respectively, were cultured in in MEGM Bullet kit (Lonza). Mycoplasma contamination was examined using MycoAlert mycoplasma detection kit (Lonza). Lentiviruses and retroviruses encoding short hairpin RNAs (Sigma) or complementary DNA were stably transduced into target cells and selected using puromycin (2 mg ml^−1^). For stable transfection, pcDNA3.1 mammalian expression plasmids (G418 for selection marker) were used. iCRT14, an inhibitor for β-catenin–TCF interaction was purchased from (Santa Cruz).

### Constructs

All constructs were generated from cDNA or open reading frame sources via PCR and constructed into mammalian expression plasmids, retroviral or lentiviral vector plasmids (FLAG-pcDNA3.1, HA-pcDNA3.1, HA-pMSCV, FLAG-pMGIB and pCDH-CMV-MCS-EF1-Cop-EGFP). nt-PAF, S33Y-β-catenin mutants were constructed using PCR-based mutagenesis. Five different lentiviral plasmids encoding shPAFs (Sigma) were tested for depletion of endogenous PAF using immunoblotting. All constructs were verified using DNA sequencing.

### Gene expression analysis

For RNA extraction, cells were processed using TRIzol reagent (Invitrogen). One microgram of RNA was used for reverse transcription using SuperScript II (Invitrogen). Next, cDNAs were used for gene expression analysis using quantitative reverse transcriptase–PCR. *GAPDH* or *HPRT* were used as internal controls for normalization. Fold induction was quantified using 2^−ΔΔCt^ methods. The primer sequences can be found in Supplementary Table 3.

### Immunoblotting

Whole-cell lysates of mammalian cells were prepared using NP-40 lysis buffer (0.5% NP-40, 1.5 mM MgCl_2_, 25 mM HEPES, 150 mM KCl, 10% glycerol, 1 mM phenylmethylsulfonyl fluoride, 12.7 mM benzamidine HCl, 0.2 mM aprotinin, 0.5 mM leupeptin and 0.1 mM pepstatin A) for 20 min at 4 °C followed by centrifugation (14,000 r.p.m. for 10 min). Supernatants were denatured in 5 × SDS sample buffer (200 mM Tris-HCl pH 6.8, 40% glycerol, 8% SDS, 200 mM dithiothreitol and 0.08% bromophenol blue) at 95 °C for 5 min followed by SDS–PAGE. For immunoblot blocking and antibody incubation, 0.1% non-fat dry milk in Tris-buffered saline and Tween-20 (25 mM Tris-HCl pH 8.0, 125 mM NaCl and 0.5% Tween-20) was used. SuperSignal West Pico and Femto reagents (Pierce) were used to detect horseradish peroxidase-conjugated secondary antibodies. The following antibodies were used for immunoblotting: PAF (Abcam (ab56773; 1:5,000) and Santa Cruz (FL-111; 1:2,000)), tubulin (Santa Cruz (A-6); 1:5,000), N-cadherin (BD Biosciences (clone 32); 1:2,000), Fibronectin (BD Biosciences (clone 10); 1:2,000), E-cadherin (Cell Signaling (VU1D9); 1:1,000), Vimentin (BD Biosciences (RV202); 1:2,000) and FLAG (Sigma (M2); 1:5,000). The original immunoblotting images can be found in Supplementary Fig. 14.

### Mammosphere and tumour sphere formation assay

76NF2V human MECs were plated in triplicate in ultra-low attachment plates (500 cells per ml) and grown in MEGM medium containing 1.3% methylcellulose (Stem Cell Technologies) supplemented with 20 ng ml^−1^ epidermal growth factor, 10 ng ml^−1^ basic fibroblast growth factor (Sigma) and B27 (Gibco). In subsequent passage, spheres were dissociated into a single cell and plated (76NF2V: 500 cells per ml and breast cancer cell lines: 20,000 cells per ml). Sphere-forming activity was assessed 2 weeks after seeding. Size of spheres was quantified using and AxioVision software (Zeiss).

### Migration and invasion assay

Cells were seeded in to 24-well cell culture inserts with 8 μm pores (BD Falcon) coated with Matrigel. After 24 h, the cells on the upper surface of the filters were removed with a cotton swab. For visualization, cells on lower filter surfaces were fixed and stained with 0.2% crystal violet. Invaded cells were analysed using Observer.Z1m microscope (Zeiss).

### Transgenic animals

*iPAF* mice previously generated were used^17^,^41^. *iPAF* pups from two independent founder strains were used for analysis (all female mice; 1–2 months of age). *PAF* transgene expression was induced by doxycycline administration in the late generations crossed with C57BL/6 mice. *Rosa26-rtTA:iPAF* or *MMTV-rtTA:iPAF* strains were administered doxycycline (2 μg ml^−1^ in 5% sucrose drinking water). *rtTA* driver mice were purchased from The Jackson Laboratory. All mice were maintained and experiments were carried out according to institutional guidelines (MD Anderson Cancer Center) and Association for Assessment and Accreditation of Laboratory Animal Care International standards.

### Xenograft assay

Nude female mice, aged 3 months, were divided into ten groups (*N*=2 each) and inoculated with 1 × 10^7^ cells of stable cell lines (MECs and MECs stably expressing PAF). After 8 weeks for adaptation, tumour development was assessed and immunohistochemistry was performed.

### Flow cytometry and fluorescent-activated cell sorting

Single-cell suspensions of each cell line were counted and incubated with antibodies: CD24-PE (BD Pharmingen (ML5; 1:100)), CD44-APC (BD-Pharmingen (G44-26; 1:100)) and EpCAM-FITC (AbD Serotec (VU-1D9; 1:800)). ALDH enzymatic activity was assessed using ALDEFLUOR kit (Stem Cell Technologies) as per the manufacturer's protocol. Diethylaminobenzaldehyde-treated sample served as a negative control for gating. FACS analysis was performed using Becton Dickinson (BD).

### Immunohistochemistry and whole-mount staining

Murine mammary tissue samples were collected and fixed in 10% formalin and processed for paraffin embedding. Sectioned samples were immunostained according to standard protocols: deparaffinization, blocking and incubation with primary and fluorescence-conjugated secondary antibodies. The following antibodies were used for immunohistochemistry: PAF (Abcam (ab56773; 1:250) and Santa Cruz Biotechnology (FL-111; 1:250)), CD44 (Abcam (IM7; 1:250)) and Ki67 (Abcam (SP6; 1:500)). For breast cancer tissue microarray, tissue microarray slides were purchased from Biomax and immunostained for PAF using anti-PAF antibody (Abcam (ab56773; 1:250)). Haematoxylin was used for nuclear counterstaining. For whole-mount mammary gland staining, mammary glands were dissected out and fixed on glass slide with Carnoy's solution (glacial acetic acid:chloroform:ethanol, 1:3:6) overnight at room temperature. Next, the glands were rehydrated and stained in aluminum carmine. The glands were then dehydrated, cleared with xylene and mounted. For breast cancer tissue analysis, breast cancer tissue microarray (Biomax; T085) was processed for nuclear antigen retrieval and the subsequent immunostaining.

### ChIP assay

Cells were cross-linked with 1% formaldehyde for 15 min at room temperature. Formaldehyde was quenched by adding glycine (final concentration, 0.125 M). After washing the cells with cold 1 × PBS solution, we harvested the cells with lysis buffer (0.5% NP40, HEPES 25 mM, KCl 150 mM, MgCl_2_ 1.5 mM, 10% glycerol and KOH pH 7.5) containing proteinase inhibitors and further incubated the cells on ice for 15 min. Cell lysates were centrifuged (5,000 r.p.m., 5 min) and supernatants were discarded. Cell lysates were subjected to sonication with ChIP–radioimmunoprecipitation assay lysis buffer (Tris 50 mM pH  8.0, NaCl 150 mM, 0.1% SDS, 0.5% deoxycholate, 1% NP40 and EDTA 1 mM; 10 times, 30 s on and 30 s off) and were centrifuged (13,200 r.p.m., 30 min). Supernatant from lysates was immunoprecipitated with antibody overnight at 4 °C and was pulled down using protein A/G PLUS-Agarose (Santa Cruz Biotechnology) by centrifugation (3,400 r.p.m., 2 min). Immunoprecipitates were further washed serially with ChIP–radioimmunoprecipitation assay, high salt (Tris 50 mM pH 8.0, NaCl 500 mM, 0.1% SDS, 0.5% deoxycholate, 1% NP40 and EDTA 1 mM), LiCl wash buffer (Tris 50 mM pH 8.0, EDTA 1 mM, LiCl 250 mM, 1% NP40 and 0.5% deoxycholate) and Tris-EDTA buffer. Finally, immunoprecipitate cross-linking was reversed by incubation at 65 °C overnight and immunoprecipitates were treated with RNase A and proteinase K to extract DNA. The following antibodies were used: RNA Polymerase II (EMD Millipore (CTD4H8; 1:100)), Oct4 (Cell Signaling (2750; 1:100) and Nanog (Cell Signaling (D73G4; 1:100)). ChIP amplicons (1–7) were detected via ChIP–PCR. Primer sequences can be found in Supplementary Table 3.

### Somatic targeting of *PAF* alleles

KO cells were generated using the CRISPR. The lentiviral plasmid contains two expression cassettes, hSpCas9 and the chimeric guide RNA , where oligos were cloned, based on the protospacer adjacent motif on the target site, using standard cloning methodology generating lentiCRISPR. HEK293T cells were transfected with packaging plasmids including the lentiCRISPR, pCMV-ΔR8.2 dVPR and pCMV-VSVG for lentiviral packaging. MDA-MB-231 breast cancer cell line was then transduced with lentiviruses and selected in puromycin for 72 h. KO was confirmed by immunoblotting and genomic DNA sequencing. Guide RNA sequences were as follows: #1: 5′- TGAAACTGATGTCGAATTAG -3′, #2: 5′- GAGTTGGGCGCACGC AAACG -3 and #3: 5- GGAGTTGGGCGCACGCAAAC -3′

### Mammary fat pad transplantation assay

Limiting dilution of MDA-MB-231 cells (*PAF* wild-type and KO) resuspended in 20 μl PBS was injected into the cleared mammary fat pads of 3-week-old NOD/SCID mice. After 12 week posttransplantation, mammary glands were harvested and positive reconstitution was determined. The frequency of SCs in the transplanted cell population was calculated using the Extreme Limiting Dilution Analysis Program (http://bioinf.wehi.edu.au/software/elda/index.html).

### Statistical analysis

The Student's *t*-test was used for comparisons of two samples. Calculation of averages was performed using at least three biological replicates. *P*-values <0.05 were considered significant. Error bars in all figures indicate s.e.m.

## Additional information

**How to cite this article:** Wang, X. *et al*. PAF-Wnt signalling-induced cell plasticity is required for maintenance of breast cancer cell stemness. *Nat. Commun.* 7:10633 doi: 10.1038/ncomms10633 (2016).

## Supplementary Material

Supplementary Figures and Supplementary References Supplementary Figures 1-13 and Supplementary References.

Supplementary Data 1 Computational analysis of PAF promoter using MatInspector software (genomatix.de), human PAF proximal promoter (-1 kb) was analyzed with default options for identification of candidate transcription factors.

Supplementary Data 2 A list of clinical cancer drugs used for PAF targeting screening clinical cancer drugs used in screening were listed.
