# Supplementary material for: PAF-Wnt signaling-induced cell plasticity is required for maintenance of breast cancer cell stemness
Source: Nat Commun. 2016 Feb 4;7:10633. doi: 10.1038/ncomms10633 (PMC4743006; doi:10.1038/ncomms10633)
Supplement: Supplementary Figures and Supplementary References — Supplementary Figures 1-13 and Supplementary References. [file ncomms10633-s1.pdf]

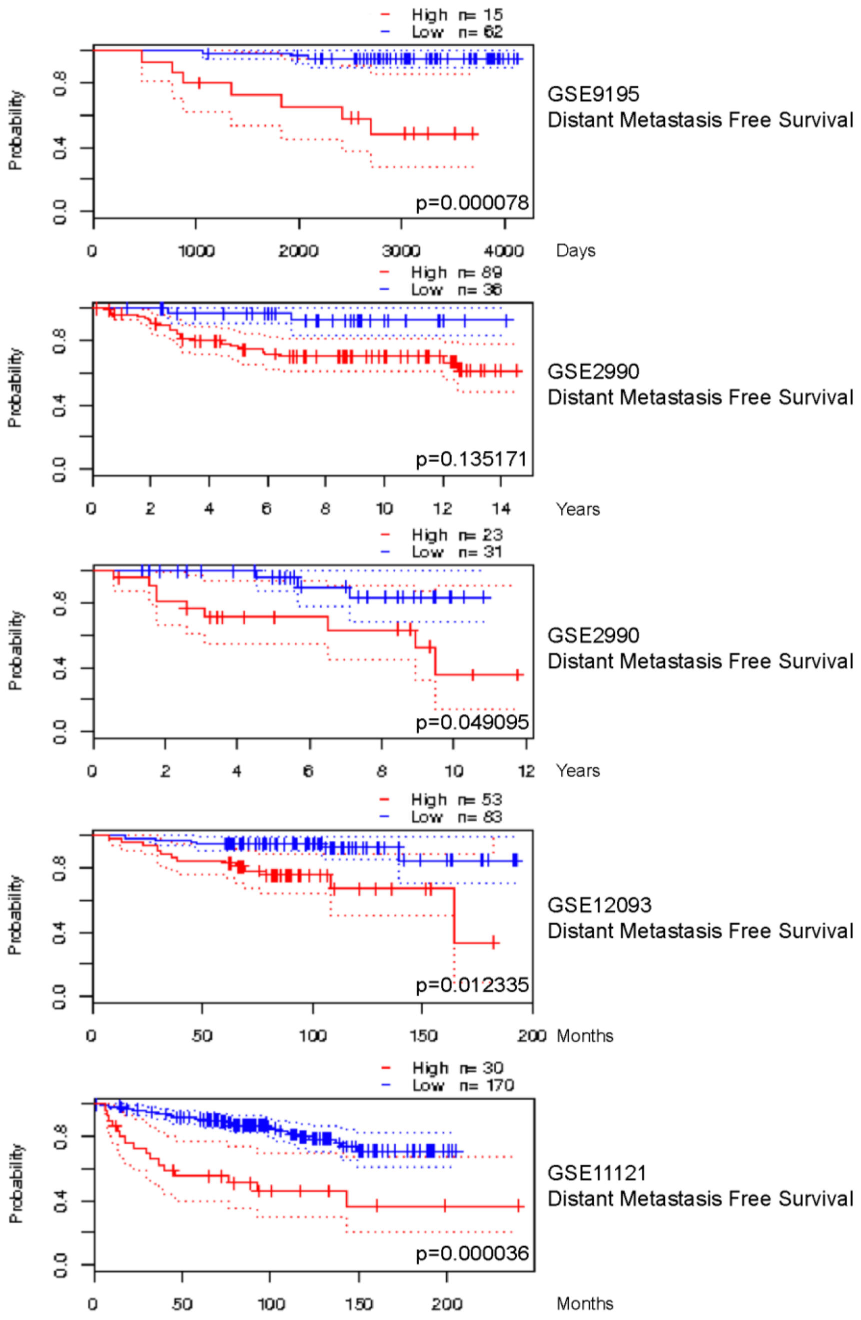

Supplementary Figure 1. Survival graphs of breast cancer based on PAF expression. Using publicly available database (Prognoscan; www.prognoscan.org), distant metastasis free survival curves were analyzed by PAF expression.

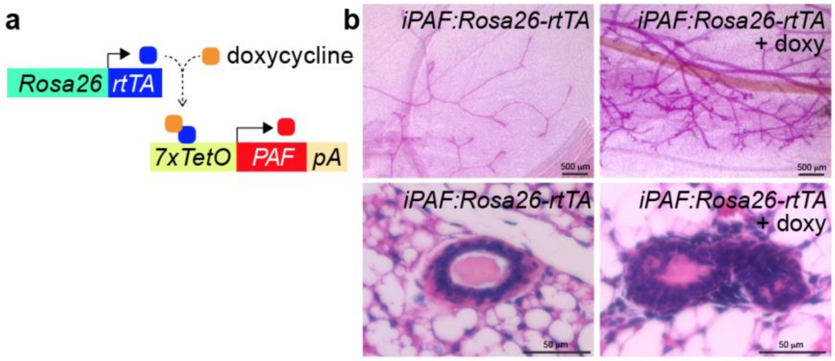

Supplementary Figure 2. Mammary ductal hyperplasia by conditional expression of PAF. *Rosa26-rtTA* driver-induced PAF expression (by doxycycline (2 mg/ml for 30 days after weaning) also induces mammary ductal hyperplasia. Of note is that ductal branching is significantly increased, compared to that of *Rosa26-rtTA*-induced *PAF*. (a) Schematic diagram of PAF conditional expression by doxycycline; (b) H&E staining of mammary ducts.

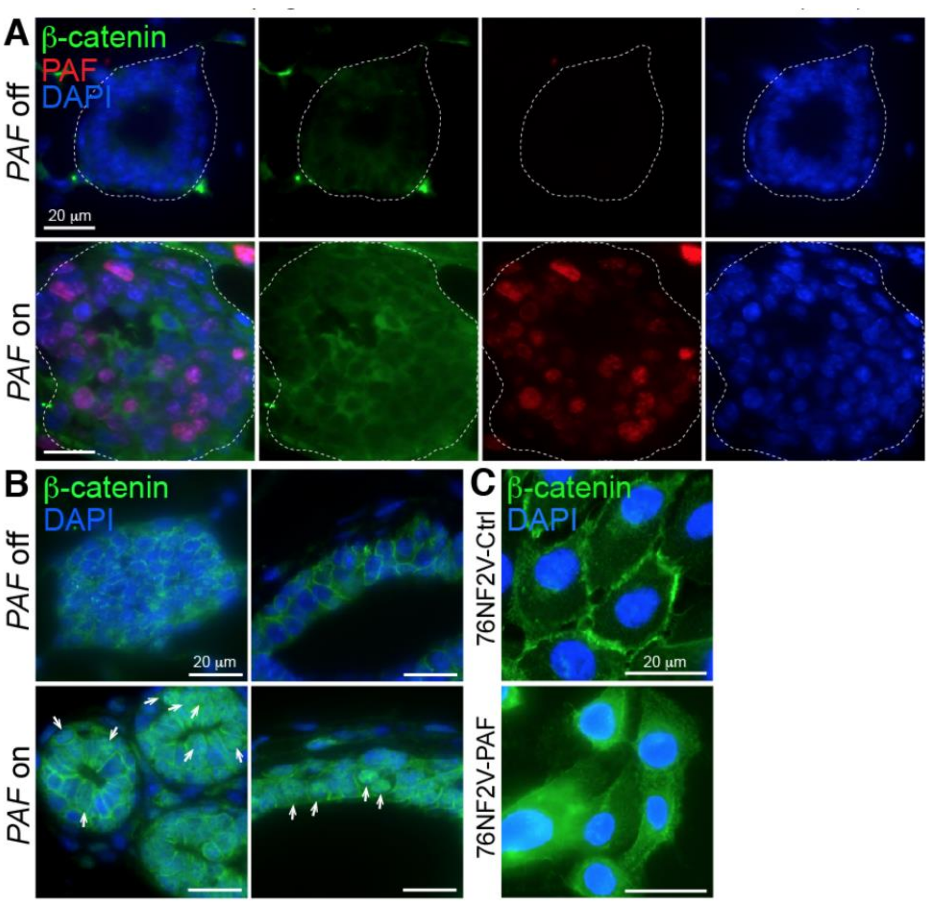

Supplementary Figure 3. Upregulation of β-catenin by conditional expression of PAF. (A) PAF-induced mammary duct was immunostained for β-catenin. Compared to control (PAF off) mice, PAF-induced (7 days) mammary ducts showed the upregulation of β-catenin. (B) Nuclear translocation of β-catenin by PAF. 1 month after PAF induction (by doxycycline administration), mammary ducts were immunostained for β-catenin. Arrows indicate the nuclear β-catenin.

(C) Upregulation of β-catenin by PAF expression. 76NF2V cells were stably transduced with retroviruses encoding β-catenin, and analyzed for IF staining for β-catenin.

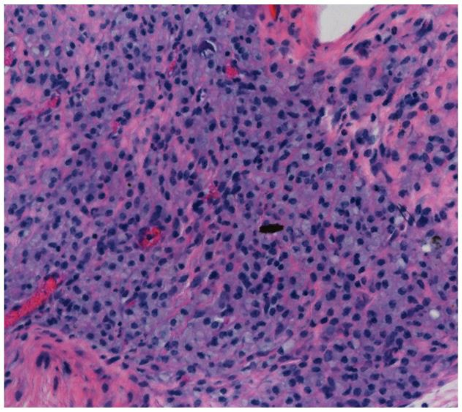

Cell line	Xenograft
76NF2V	No tumor
76NE7	No tumor
76NE6	No tumor
76NE6/TERT	No tumor
HMLE	No tumor
76NF2V-PAF	No tumor
76NE7-PAF	No tumor
76NE6-PAF	Tumor
76NE6/TERT-PAF	Tumor
HMLE-PAF	No tumor

Supplementary Figure 4. *Ex vivo* tumor formation by PAF and p53 inactivation. Xenograft transplantation of mammary epithelial cells stably expressing PAF. 8 weeks after subcutaneous injection of cells, tumors were collected for immunohistochemistry (hematoxylin & eosin staining). 76NF2E7 or 76NF2R6 cells are stably expressing E7 or E6 proteins, respectively. 76NE6/TERT cells express both E6 and TERT. Of note, 76NE6 cells stably expressing PAF develop *ex vivo*, implying that p53 inactivation might be required for tumor initiation in the setting of PAF-Wnt signaling activation.

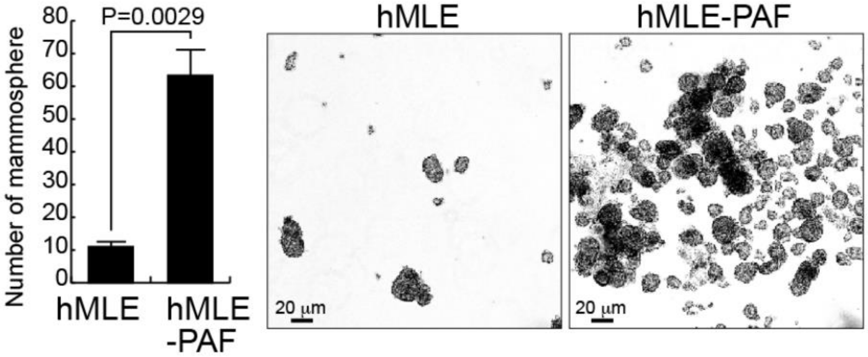

Supplementary Figure 5. Increased mammosphere formation by ectopic expression of PAF. hMLEs were stably transduced with retrovirus encoding PAF. Mammosphere was analyzed (left: quantification; right: the representative images). N=3; Student *t*-test; error bars=SEM.

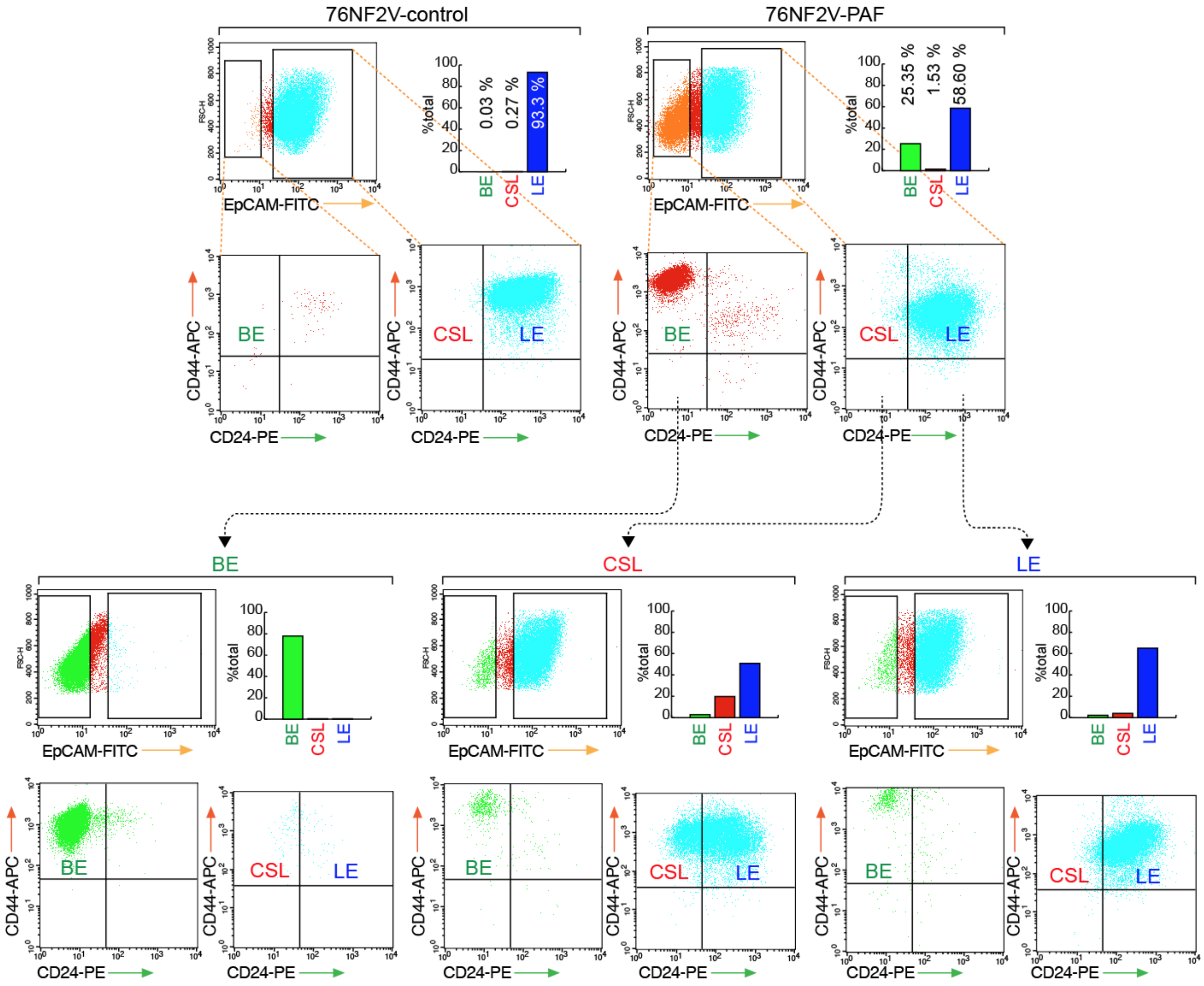

Supplementary Figure 6. PAF-induced cell heterogeneity.
Each group of cells (SC, LE, and BE) from 76NF2V-PAF cells was sorted using FACS and cultured for 6 days. Then, cells were reanalyzed for cell properties using FACS (CD24, CD44, and EpCAM).

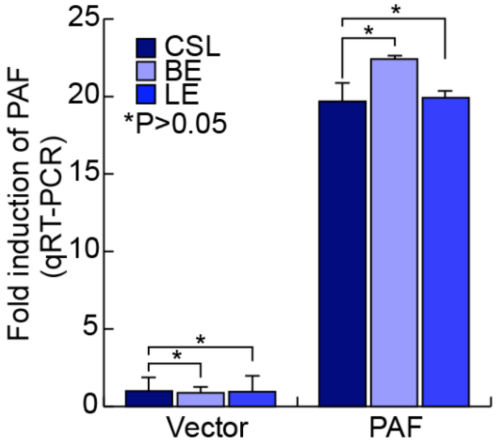

Supplementary Figure 7. Expression of *PAF* in 76NF2V-PAF cells. CSL, BE, and LE cells were sorted using FACS. Then, PAF expression was examined by qRT-PCR. N=3; Student *t*-test; error bars=SEM.

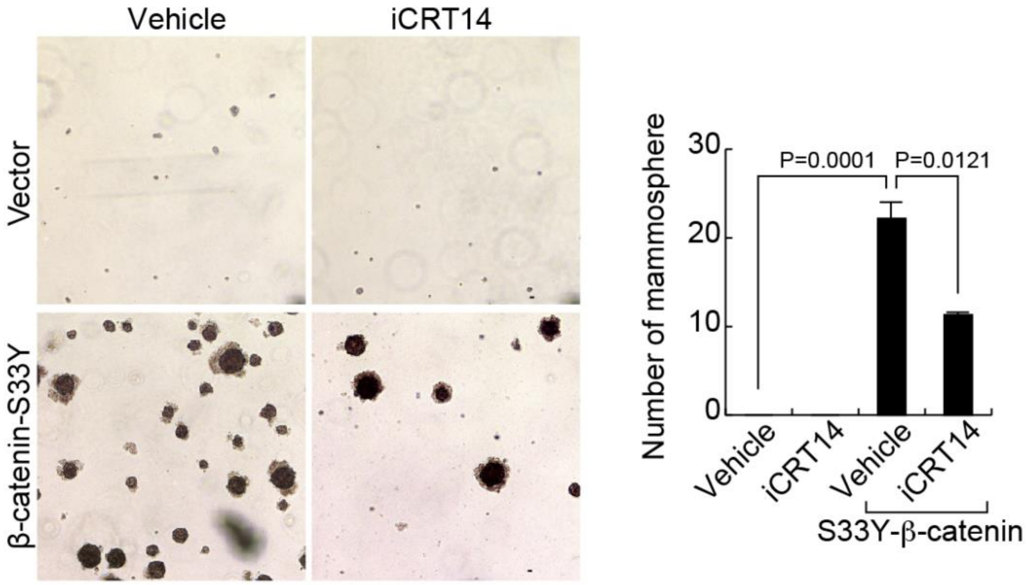

Supplementary Figure 8. Inhibition of β-catenin-induced generation of CSL by iCRT14. 76NF2V cells stably expressing S33Y β-catenin were treated with iCRT14, and examined for mammosphere formation (left: the representative images; right: quantitative analysis). N=3; Student *t*-test; error bars=SEM.

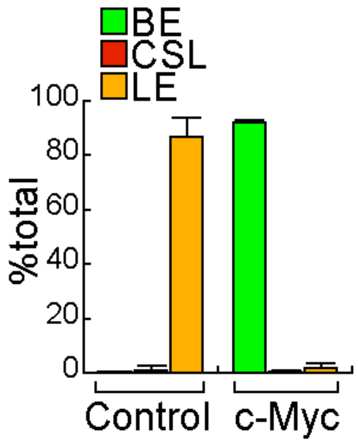

Supplementary Figure 9. Myc-induced EMT does not generate CSL cells.
Using retrovirus encoding c-Myc, 76NF2V-c-Myc stable cells were established and analyzed for CD24, CD44, and EpCAM expression using FACS. N=3; Student *t*-test; error bars=SEM.

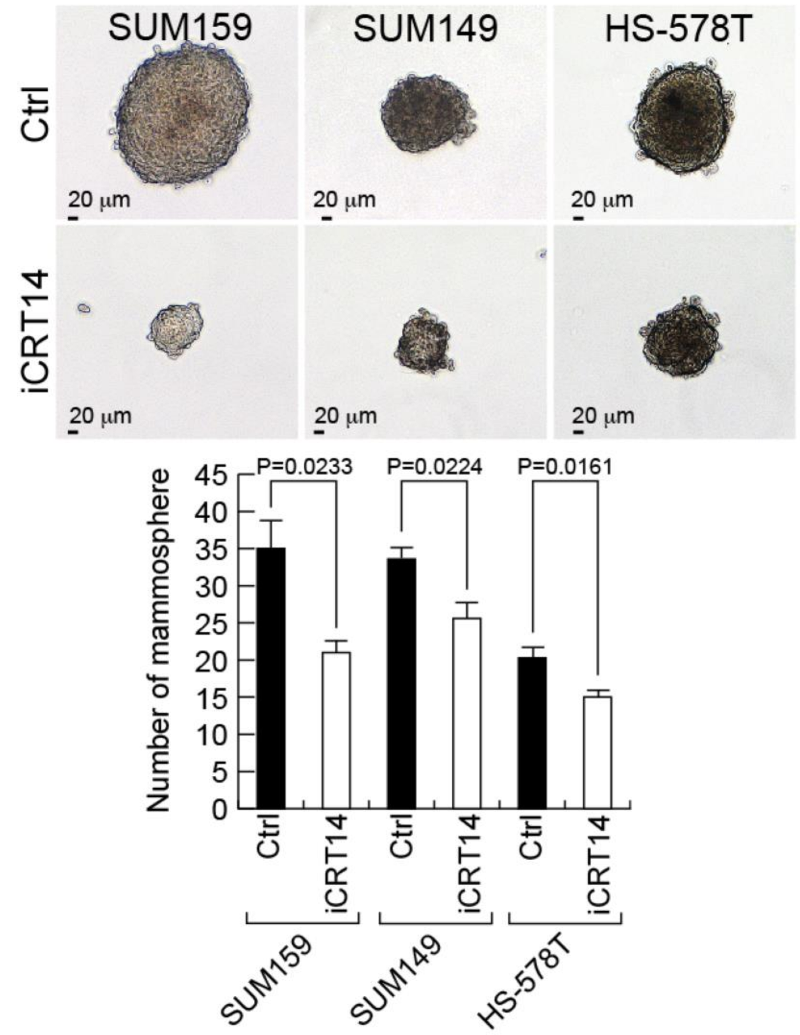

Supplementary Figure 10. iCRT14 inhibits mammosphere formation. Breast cancer cell lines were treated with iCRT14 during mammosphere formation assays (upper: the representative images; lower: quantitative analysis). N=3; Student *t*-test; error bars=SEM.

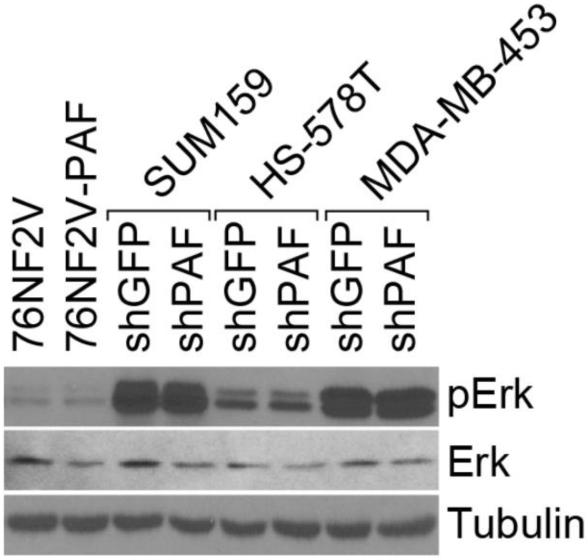

Supplementary Figure 11. No changes in MAPK signaling by PAF depletion. Breast cancer cells lines stably expressing shGFP (control) and shPAF were analyzed by IB for phospho Erk. 76NF2V cells stably expressing PAF also do not show upregulation of phosphor Erk.

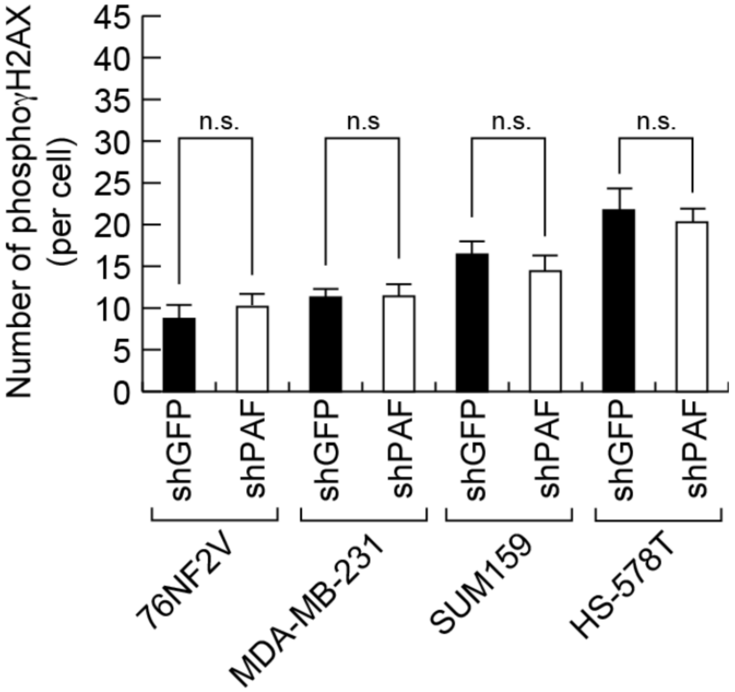

Supplementary Figure 12. No changes in DNA double strand break in the depletion of PAF. Breast cancer cells lines stably expressing shGFP (control) and shPAF were analyzed by IF staining for phospho gamma H2AX, a surrogate marker for DNA DSBs. 76NF2V cells served as a negative control. N=3; Student *t*-test; error bars=SEM.

Cell line	Assays	Results by NVP-AUY922 treatment	References
MDA-MB-231	In vitro treatment	Activation of Caspase-3/7 was not changed, but cell death was induced	Eccles (2008), Jensen (2008)
MDA-MB-231	Spheroid growth	Reduced sphere growth	Pamienta (2011)
MDA-MB-231	Xenograft	Reduced tumor growth	Terwisscha (2014)
BT474	In vitro treatment	Rapid activation of Caspase-3/7, and cell death was induced	Eccles (2008), Jensen (2008)
BT474	Xenograft	Regression of tumor volume	Eccles (2008), Jensen (2008)
BT474	Spheroid growth	Reduced sphere growth	Bergstrom (2008), Monazzam (2009)
SKBR3	In vitro treatment	Reduced cell growth	Eccles (2008), Jensen (2008)
MDA-MB-157	In vitro treatment	Reduced cell growth	Jensen (2008)
BT20	In vitro treatment	Reduced cell growth	Jensen (2008)
MDA-MB-468	In vitro treatment	Reduced cell growth	Jensen (2008)
MDA-MB-468	Xenograft	Reduced tumor growth	Chen (2014)
MCF-7	In vitro treatment	Reduced cell growth	Jensen (2008)

Trial	Results	References
Phase I	Acceptable tolerability	Sessa (2013)
Phase II	Undergoing	Sessa (2013), Zagouri (2013)
Patient treatment	Tumor regression	Gaykema (2014)

Supplementary Figure 13. Inhibition of breast cancer cell proliferation by NVP-AUY922. In vitro, ex vivo, and clinical applications of NVP-AUY922 as breast cancer treatment are shown. the accumulating literatures using NVP-AUY922 as a drug for breast and other cancers (**Fig. R2.3**) [1-8].

Supplementary References

1. Jensen, M.R., *et al.* NVP-AUY922: a small molecule HSP90 inhibitor with potent antitumor activity in preclinical breast cancer models. *Breast cancer research : BCR* 10, R33 (2008).
2. Gaykema, S.B., *et al.* 89Zr-trastuzumab and 89Zr-bevacizumab PET to evaluate the effect of the HSP90 inhibitor NVP-AUY922 in metastatic breast cancer patients. *Clinical cancer research : an official journal of the American Association for Cancer Research* 20, 3945-3954 (2014).
3. Chen, S.M., *et al.* HSP90 inhibitor AUY922 abrogates up-regulation of RTKs by mTOR inhibitor AZD8055 and potentiates its antiproliferative activity in human breast cancer. *International journal of cancer. Journal international du cancer* 135, 2462-2474 (2014).
4. Sessa, C., *et al.* First-in-human phase I dose-escalation study of the HSP90 inhibitor AUY922 in patients with advanced solid tumors. *Clinical cancer research : an official journal of the American Association for Cancer Research* 19, 3671-3680 (2013).
5. Wainberg, Z.A., *et al.* Inhibition of HSP90 with AUY922 induces synergy in HER2-amplified trastuzumab-resistant breast and gastric cancer. *Molecular cancer therapeutics* 12, 509-519 (2013).
6. Pimienta, G., Herbert, K.M. & Regan, L. A compound that inhibits the HOP-Hsp90 complex formation and has unique killing effects in breast cancer cell lines. *Molecular pharmaceutics* 8, 2252-2261 (2011).
7. Oude Munnink, T.H., *et al.* (89)Zr-trastuzumab PET visualises HER2 downregulation by the HSP90 inhibitor NVP-AUY922 in a human tumour xenograft. *European journal of cancer* 46, 678-684 (2010).
8. Eccles, S.A., *et al.* NVP-AUY922: a novel heat shock protein 90 inhibitor active against xenograft tumor growth, angiogenesis, and metastasis. *Cancer research* 68, 2850-2860 (2008).
